# The Contribution of Rat Studies to Current Knowledge of Major Depressive Disorder: Results From Citation Analysis

**DOI:** 10.3389/fpsyg.2020.01486

**Published:** 2020-07-14

**Authors:** Constança Carvalho, Filipa Peste, Tiago A. Marques, Andrew Knight, Luís M. Vicente

**Affiliations:** ^1^Centro de Filosofia das Ciências da Universidade de Lisboa (CFCUL), Faculdade de Ciências da Universidade de Lisboa, Lisbon, Portugal; ^2^Centre for Environmental and Marine Studies, Departamento de Biologia, Universidade de Aveiro, Aveiro, Portugal; ^3^Centre for Research into Ecological and Environmental Modelling, Departamento de Biologia Animal, Centro de Estatística e Aplicações, Faculdade de Ciências, Universidade de Lisboa, Lisbon, Portugal; ^4^Centre for Animal Welfare, University of Winchester, Winchester, United Kingdom

**Keywords:** major depressive disorder, animal models, animal use alternatives, citation analysis, rat

## Abstract

Major depressive disorder (MDD) is the most severe depression type and one of the leading causes of morbidity worldwide. Animal models are widely used to understand MDD etiology, pathogenesis, and treatment, but the efficacy of this research for patients has barely been systematically evaluated. Such evaluation is important given the resource consumption and ethical concerns incurred by animal use. We used the citation tracking facilities within Web of Science and Scopus to locate citations of original research papers on rats related to MDD published prior to 2013—to allow adequate time for citations—identified in PubMed and Scopus by relevant search terms. Resulting citations were thematically coded in eight categories, and descriptive statistics were calculated. 178 publications describing relevant rat studies were identified. They were cited 8,712 times. More than half (4,633) of their citations were by other animal studies. 794 (less than 10%) were by human medical papers. Citation analysis indicates that rat model research has contributed very little to the contemporary clinical understanding of MDD. This suggests a misuse of limited funding hence supporting a change in allocation of research and development funds targeting this disorder to maximise benefits for patients.

## Introduction

Depression is the leading cause of disability worldwide (World Health Organization, 2019). Nowadays, it is judged to affect more than 320 million people of all ages and genders (Vos et al., 2016), even though it is more frequent in women than man (Ferrari et al., 2013).

Currently, and according to DSM-5 (American Psychiatric Association, 2013), there are eight main forms of depression: major depressive disorder (MDD), persistent depressive disorder, premenstrual dysphoric disorder, disruptive mood dysregulation disorder, substance/medication-induced depressive disorder, depressive disorder due to another medical condition, non specified depressive disorder, and other specified depressive disorder. MDD is the most severe, prevalent, and disabling depression type (Malhi and Mann, 2018). It is characterized by a persistent depressed mood or loss of pleasure, along with four out of the following symptoms: significant weight loss or gain; insomnia or hypersomnia; psychomotor agitation or retardation; fatigue or loss of energy; feelings of worthlessness or excessive or inappropriate guilt; diminished ability to think, concentrate, or make decisions; recurrent thoughts of death (American Psychiatric Association, 2013). For MDD to be diagnosed, the patient needs to fulfill five diagnostic criteria out of a pool of nine, which means that the same disorder may present differently in different subjects (Kaufman, 2018). The variety of both symptoms and biomarkers has led to the recent suggestion that there might be several subtypes of MDD (Beijers et al., 2019).

MDD etiology is not completely understood yet. Most authors agree that there is a combination of biological and environmental factors that determine the triggering of the disorder (Mandelli and Serretti, 2013). Biological factors to take into account include genes, neurotransmitters, and hormones, while environmental factors include childhood trauma, stressful life events, sexual abuse, low educational attainment, and differences in personality traits.

Evidence suggests that there are genetic factors involved, but even though more than 100 candidate genes have been investigated, a clear connection between specific genes and MDD has not yet been established (Shadrina et al., 2018). Furthermore, studies suggest that variations in different genes, each with a minor effect, combine to increase the risk of developing this disorder (Wray et al., 2018). Most studies suggest that MDD patients have imbalanced brain chemistry at neurotransmitters level (Beijers et al., 2019). Among those, the majority of the studies indicate dopamine, norepinephrine, and serotonin as the most implicated in MDD etiology (Belujon and Grace, 2017). Others stress the involvement of glutamate (Réus et al., 2018). Nonetheless, some studies find no differences in neurotransmitters between MDD patients and healthy controls (for a review, see Beijers et al., 2019). Similarly, it is widely accepted that hormones play a role in MDD etiology (Druzhkova et al., 2019) but there is no clear-cut connection between a specific hormone secretion and MDD. For example, Asadikaram et al. (2019) found differences between MDD patients and healthy controls in hormone levels of adrenocorticotropic hormone, testosterone, thyroid–stimulating hormone, free thyroxine index, and cortisol/dehydroepiandrosterone sulfate (DHEA–S), while others consider vasopressin and oxytocin to play a pivotal role in MDD etiology (for a review, see Iovino et al., 2018).

Recent studies also suggest that there is a link between inflammation and MDD, suggesting that MDD has an inflammatory subtype (Beijers et al., 2019), but the claims that inflammation has a role in etiology of MDD are still being disputed (Miller, 2018). The same happens with changes in gut microbiome in MDD patients (Winter et al., 2018). While the link between gut microbiome and depression is well documented, the question of the causality in the connection between the two remains to be robustly answered (Winter et al., 2018). It is important to mention that these patterns may be true for all biological changes found in MDD patients. It is almost impossible to determine if the biological changes caused MDD or if MDD caused the biological changes. Conversely, most environmental factors involved in MDD are definitely a primary cause. In this regard, the big unanswered question that remains is why does the same life event trigger MDD in one person and not in another.

Among the most documented environmental factors linked to MDD are childhood traumas, which also cause biological changes in the brain of MDD patients (Yu et al., 2019), stressful life events, sexual abuse, low educational attainment (Peyrot et al., 2013), and personality traits (Bensaeed et al., 2014). Other disorders and traits are also strong predictors for MDD. For example, a big longitudinal study showed that people who present anxiety traits in their twenties are more prone to develop MDD in their thirties (Gustavson et al., 2018). Also Parkinson’s, Migrains’, Alzheimer’s patients, among others, have high prevalence of MDD (Ketharanathan et al., 2014; Muneer et al., 2018; Tao et al., 2019).

It is not always possible to determine a proximal cause. MDD may have seasonal or peri-partum onset, as well as being induced by other disorders (e.g., Parkinson’s) or substance ingestion, but it can also emerge without an obvious reason (American Psychiatric Association, 2013).

Major depressive disorder pathogenesis is as diverse as its etiology. Even though there are several different treatment courses available (for a review, see for example Pandarakalam, 2018), 50–60% of patients develop treatment resistant depression, i.e., do not enter remission even after trying different courses of treatment (Kraus et al., 2019), and only 52% of patients achieve a full recovery (Novick et al., 2017).

Due to its complexity, MDD is particularly hard to study, but its severity, prevalence, and significant economic burden make it a moral and social imperative to keep investing this research field. Yet, its research funding has been scarce when compared to other disorders (e.g., cancer) (Ledford, 2014).

Randomized controlled trials (RCTs) are considered to be the gold standard for empirical research (Hariton and Locascio, 2018), namely, in MDD’s potential treatments and interventions (Monsour et al., 2019) but they are unsuitable for all purposes. Some authors consider observational longitudinal studies to be more useful in understanding the etiology and pathogenesis of human disorders (Frieden, 2017) pointing out that they also overcome ethical and practical limitations of RCTs such as the insufficient study duration or the disregard of unpredictable variables that affect patients in their daily lives (Song and Chung, 2010). Others stress the importance of basic and applied research aiming to understand MDD’s mechanisms in a controlled environment (e.g., Papassotiropoulos and De Quervain, 2015).

In this regard, advanced magnetic resonance imaging techniques used in patients and healthy controls can be a powerful tool regarding the physiological and metabolic characterization of brain tissue, in the same way that single photon emission computed tomography and positron emission tomography imaging modalities provide valuable data on brain function and activity (Tsougos et al., 2019).

Post-mortem studies as well as cell-based disease modeling are another valuable set of tools in understanding the biology of psychiatric disorders. As cellular biology techniques evolve new ways to generate and preserve human cells *in vitro* emerge (e.g., induced pluripotent stem cells, trans differentiation technologies for deriving neurons from adult humans, Vadodaria et al., 2018). However, they are insufficient to fully understand the pathways and progression of complex disorders. Some authors assert that systems biology might be the answer as it can integrate and model different levels of human experimental data—molecular, cellular, tissue, organ, clinical, and population disorders (Langley, 2014). Others suggest a mind-brain paradigm that combines simultaneous use of imaging techniques with the use of scales and/or other tests validated for psychodiagnostics as a way to build a bridge between neuroscience and psychological sciences to study mental disorders (Stoyanov, 2009). Others claim that the only way to overcome such limitations is by resorting to animal models, which are seen by some as crucial for MDD research (e.g., Wang et al., 2017; Akil et al., 2018), despite their well-recognized limitations with respect to human predictability (Akil et al., 2018).

To overcome these limitations, combinations of different animal models are proposed (Akil et al., 2018), different transgenic lines of rats are generated (as described by Bailey, 2019) and efforts are made to overcome the biological differences between species that keep emerging as extrapolation barriers (Hodge et al., 2019). All the above involve high economic costs and consume a tremendous amount of animal lives. The reason behind this is because it is assumed that animal use is unavoidable and its withdrawal would jeopardize human health. However, very few studies have addressed the contribution of animal models to MDD research through significant critical scrutiny within peer-reviewed literature. Specifically, to our knowledge, the contribution of rats for this aim has never been evaluated in such terms, even though rodents are undoubtedly the most frequently used animals regarding this context. Even though mice are by far the most used rodents in biomedical research, an initial search in PubMed, a search engine that comprises more than 30 million biomedical literature publications, indicated that species within genus Rattus were highly used in MDD research, which made them an interesting case study. To evaluate the contribution of animal models to MDD research is important for ethical and economic reasons. As a society, we should make an informed decision on whether we should proceed improving animal models until we find a suitable one or if we should halt the current paradigm and invest more in other methods that might be more promising as well as cheaper and less ethically contentious (Carvalho et al., 2019).

To conduct such evaluation, we performed a citation analysis on original publications describing rat data within MDD research. A citation analysis as defined by Garfield and Merton (1979) consists of determining the number of citations target papers (in this case original papers using rat models to study MDD) receive, as well as determining citation patterns—in this case which sort of papers are citing the target papers (e.g., animal research papers, human research papers, review papers). Granting that the studies cited guide and influence authors’ work (Burright et al., 2005) and that citation level has been related to clinical relevance in the past (Pound and Nicol, 2018), such citation analysis can be used as an indicator to evaluate the contribution of rat studies to current clinical knowledge in MDD, as has been done for other disorders (e.g., Knight, 2007; Long et al., 2014; Carvalho et al., 2016) as well as for other species in regard to MDD research (Carvalho et al., 2019).

If rat studies are informing the human medical research community, then we would expect that:

1. Most of the papers would be cited at least once in subsequent human medical papers;
2. The proportion of citations by human medical papers would be substantially higher when compared to other research categories.

## Methods

The citation analysis was performed between January and August of 2019. PubMed and SCOPUS were searched for publications using rat models to investigate MDD. We searched PubMed using Medical Subject Heading search terms (MeSH terms): “Depressive Disorder, Major” AND “rat” OR “rodent.” MeSH terms are a comprehensive list of key terms made available by PubMed designed to identify all relevant studies in an area (Uman, 2011). So, searching for “MDD” retrieves other nomenclatures for the same disorder such as melancholia. Similarly, the search term “rat” retrieves papers using all rat species. We used PubMed filters to exclude review articles (“review,” “systematic review,” “meta-analysis,” “bibliography”) as well as opinion articles (“biography,” “auto-biography,” “comment,” “editorial,” “interview”). Since Scopus does not have the MeSH term tool, we used the search terms “MDD” AND (“rat” OR “rattus”) in the search fields. We included journal papers, books, research reports, and conference proceedings written in English or Portuguese, which are within our language proficiencies. We restricted our search to publications prior to 31 December 2013, to allow adequate time for citation of articles to occur. We did not include a lower date limit.

Since our goal was to evaluate the contribution of animal models—particularly rat models—to current clinical knowledge of MDD, we manually excluded from our analysis all the papers that reported animal and human data, as well as papers reporting other species’ data (e.g., mice). Hence, we included only original papers focused on MDD, exclusively using data obtained from rats. We excluded papers describing other disorders, data from other species along with data from rats, and papers that did not present first hand data (e.g., review papers). Out of 237 papers originally located through the search engines’ filters, we manually excluded 59 because they met exclusion criteria.

The retrieved papers were subjected to a subsequent citation analysis using the cited reference search facility within Scopus and Web of Science.

Web of Science is a major scientific citation indexing service that encompasses over 50,000 scholarly books, 12,000 journals, and 160,000 conference proceedings. Scopus is the largest citation database; it covers nearly 36,377 titles from approximately 11,678 publishers.

For each rat study, we recorded the total number of times it was cited, and allocated each citation to one or more of seven categories, defined prospectively:

– Animals. This category included all animal studies from observational ethological studies to invasive procedures as defined by Knight (2011), i.e., interfering with bodily integrity (whether through puncture or incision) or production of genetically modified animals. This category also included severe procedures (as defined by current European Legislation Directive 2010/63/EU) commonly used in mental disorder research such as inescapable electroshock or isolating social animals for long periods. We recorded within this category which animal papers focused on MDD and which focused on other subjects.
– Humans. This category included papers that used human participants. They included clinical or treatment trials (either drug trials or non-pharmacological treatments), papers aiming to explore psychological, social, biochemical, physiological, genetic, or neurological variables related to MDD or other human disorders; as well as papers aiming to understand the relationship between MDD and other disorders (co-morbidities) in human patients. We recorded within this category which human papers focused on MDD and which focused on other disorders.
– Reviews. This category included narrative reviews, systematic reviews, meta-analysis, as well as extensive opinion papers that did not report original empirical data. We included reviews focused on animals and on humans alike.
– Editorials. This category included editorials, comments, and clinical guides.
– *In vitro*. This category included exclusively cell-line data. Whenever the source of the tissue or cell was a human participant (either alive or post-mortem) or a laboratory animal killed for such purpose the paper was allocated into “human paper” or “animal research paper,” respectively.
– *In silico*. This category included data obtained via computer simulations of human data.
– Social. This category included human surveys or other social perception papers.

Whenever it was not possible to define the category of the citing paper (due to language barriers—i.e., papers written in a language that was not English or Portuguese—or absence of the abstract), the paper was denoted “not available” and removed from the sample. If more than one category could be assigned to a citing paper (e.g., animal research and human paper), then that paper was allocated to every appropriate category.

To evaluate if the proportions of citations made by human publications and animal publications on MDD were different, we used a *t*-test. Results were considered statistically significant when *P* < 0.05. The analyses were performed in R 3.6.1 (R Core Team, 2019).

## Results

The 178 original rat studies focused on MDD that were published before the end of 2013 were cited 8,712 times by August 2019. Of these 178, 87 (49%) studies were never cited in subsequent publications describing human studies on MDD, and 53 (30%) were never cited in any publications related to human research, either focused on MDD, or on other disorders such as post-traumatic stress disorder or bipolar disorder.

**FIGURE 1:**
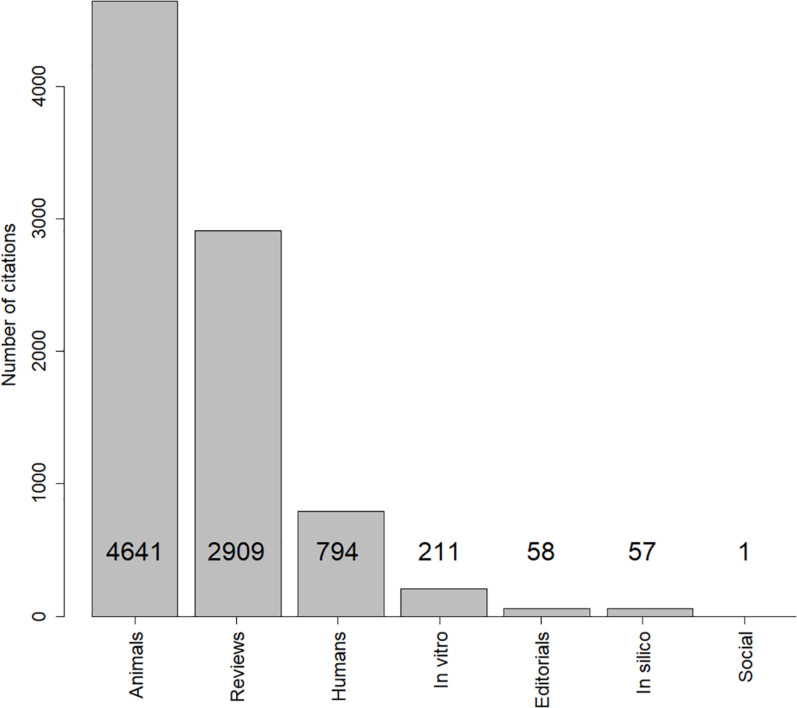
Frequency of citations by category established in this study.

As shown in Figure 1, rat studies were mainly cited by other animal research papers (4,641), followed by review papers (2,909), human studies (794), *in vitro* papers (211), editorials (58), *in silico* papers (57), and human social papers (one). 230 citations were unavailable to us due to access or language barriers. These were removed from further analysis.

The proportion of citations by human medical papers was 9.1% while the proportion of citations by animal papers was 53.3%. This corresponds to a mean difference between the proportions of citations by human and by animal papers of −46% (*p* < 0.001). Beyond the statistical significance, this is certainly a considerable practical difference that reflects almost 100^∗^(53.3–9.1)/53.3 less citations by human papers than by animal papers.

## Discussion

The majority of the rat papers located in this study were cited by subsequent animal research papers, but about half (49%) of the original papers retrieved were never cited in subsequent papers related to MDD in humans, and in fact about a third (30%) were never cited in any subsequent human studies.

Our citation analysis revealed that only around a tenth (9.1%) of the total number of citations were by human medical papers. This result contradicted the hypothesis that the proportion of citations by human medical papers would be substantially higher when compared to other research categories. This hypothesis was based on the assumption that animal models are essential to clinical research, as promulgated by several authors (e.g., Wang et al., 2017; Akil et al., 2018). Hence, our results raise doubts about the justification for these animal studies.

The results of our study are in agreement with previous studies that empirically evaluated the contribution of animal models to human healthcare either through citation analysis (e.g., Shapiro, 1998; Knight, 2007; Long et al., 2014; Carvalho et al., 2016) either through other methods such as systematic reviews (e.g., Perel et al., 2007), social science studies (e.g., Shapiro, 1998; Compton et al., 2019), historical analysis (e.g., Menache, 2012), among others (for an extensive review on this topic see, for example, Knight, 2019).

This suggests that biomedical research resorting to animal models is not normally considered significant, or particularly visible to, the human medical research community.

Supporters of animal models of human disorders claim that this happens: (a) due to differences in the way basic animal work and human clinical trials are conducted, and propose a change to a translational biomarker-based approach within early steps of pre-clinical research (Garner, 2014); or that this occurs (b) due to failings in study design, conduct, analysis, and reporting (as described by Pound and Ritskes-Hoitinga, 2018), which could be resolved with better reporting and better methodological quality (as proposed by Fabian-Jessing et al., 2018).

Opponents of the use of animal models point out that animal models lack external validity, i.e., findings derived in one setting, population, or species cannot be reliably applied to other settings, populations, and species, which is unavoidable since animal models: (a) oversimplify complex human disorders and the conditions in which they occur (Stoyanov, 2009; Pound and Ritskes-Hoitinga, 2018); (b) are unsuitable models due to species differences, proposing as a possible solution a shift toward human-based non-clinical research (Pound and Ritskes-Hoitinga, 2018).

This paradigm change toward human-based research is gaining more and more supporters both for ethical and scientific reasons (e.g., LaFollette and Shanks, 1995; Langley, 2014; Pound and Ritskes-Hoitinga, 2018; Ram, 2019). But the resistance from animal researchers in face of this shift remains significant and is evident not only in the slowness to recognize the growing body of evidence against the use of animals as models, but also in the emphasis placed on refinement of animal use (Franco and Olsson, 2014) (the third R as defined by Russell and Burch, 1959, instead of on the first and most important R—replacement with non-animal alternatives). Considering this, Frank (2005) proposed that animal experimentation constitutes a good example of path dependency, which is a well-documented phenomenon that states that what has occurred in the past persists because of resistance to change.

Our results also show that more than half (53%) of the citations our target papers received were by subsequent animal papers, which strengthens the likelihood of the path dependency phenomenon described above. It can be argued that there is a need to have a substantial amount of animal research before achieving a critical mass that can lead to useful breakthroughs in human health, which might explain the high level of citations of animal papers by subsequent animal papers. Nonetheless, it does not explain the low level of citations by human papers, especially when this does not appear to be a trend in human-based approaches (*in vitro* and *in silico*) which received more citations by human medical papers in a small study previously conducted (Carvalho et al., 2019). Furthermore, a recent citation analysis in another field of human health research also found *in vitro* papers to be the most cited papers, above reviews, and animal experimentations papers (Adnan and Ullah, 2018).

We acknowledge that this study has certain limitations. Even though we used two very large bibliographic databases to attempt to locate all publications that met our search criteria, we acknowledge that a small minority of relevant papers may not have been retrieved (e.g., due to labeling errors) or may have been excluded due to human error, as only one person selected which retrieved papers met exclusion criteria.

Similarly, we did not take note of certain citations such as self-citations, in-house citations (i.e., citations by colleagues from the same insitution), and content-irrelevant citations (e.g., the paper is cited for other reasons other than its content). Such citation types were not considered significant in several studies and were similarly dismissed in recent studies (Huang et al., 2019).

Also, we did not analyze the quality of citing papers as in previous studies (Carvalho et al., 2016, 2019). This might have resulted in an even lower, but a truer, indication of the contribution of rat models for current clinical knowledge of MDD, given that not all were likely of high quality.

Finally, we acknowledge that being cited by human medical papers is not the only indicator of the clinical usefulness of research. Uncited studies may also contribute, for example, through direct transfer of knowledge between scientists and clinicians. Most clinicians are not involved in research, i.e., they do not publish; hence, it is possible that practitioners involved in the treatment of MMD are informed by or utilize findings from rat studies through direct transfer of knowledge. A citation analysis does not measure this effect. However, it is also possible that clinicians are less aware of research results, than researchers. Thus, a survey on practitioners—which we did not perform—would have been an interesting complement to our results.

Review papers on depression that might inspire authors likely cite animal papers, nonetheless if the animal paper is really relevant for the authors’ hypothesis or method it is likely the primary paper will be cited and not solely the review paper that referred to it.

Citation rates may also be affected by factors such as article length, number of authors, their country, and university of affiliation (Leimu and Koricheva, 2005). Despite their limitations, however, citation frequencies do normally provide reasonably objective approximation of the importance of research results within a field. Research that makes a significant contribution—such as by confirming or refuting important hypotheses—is likely to be cited by subsequent papers. Research that is inconclusive or lacking in significance is much less likely to be cited.

Accordingly, we believe that our citation analysis provides a valid and critical assessment of the contribution of rat models to current clinical knowledge of MDD. Such evaluation of the efficacy of research approaches is important to ensure the most responsible allocation of research funds and scientific resources, to maximize the likelihood of clinical benefit to patients, and to minimize the consumption of animal lives in research where such benefits are unlikely.

## Data Availability Statement

The raw data supporting the conclusions of this article will be made available by the authors on request.

## Author Contributions

CC developed the study concept with the supervision of AK, TM, and LV. CC and FP performed data collection. CC and TM made data analysis. All authors contributed to the study design and the draft of the manuscript and approved the final version of the article for submission.

## Conflict of Interest

The authors declare that the research was conducted in the absence of any commercial or financial relationships that could be construed as a potential conflict of interest.
